# The Training Characteristics of Recreational-Level Triathletes: Influence on Fatigue and Health

**DOI:** 10.3390/sports9070094

**Published:** 2021-06-25

**Authors:** João Henrique Falk Neto, Eric C. Parent, Veronica Vleck, Michael D. Kennedy

**Affiliations:** 1 Athlete Health Lab, Faculty of Kinesiology, Sport, & Recreation, University of Alberta, Edmonton, AB T6G 2H9, Canada; kennedy@ualberta.ca; 2 Department of Physical Therapy, Faculty of Rehabilitation Medicine, University of Alberta, Edmonton, AB T6G 2G4, Canada; eparent@ualberta.ca; 3 CIPER, Faculdade de Motricidade Humana, Universidade de Lisboa, 1499-002 Cruz Quebrada-Dafundo, Lisboa, Portugal; vvleck@fmh.ulisboa.pt

**Keywords:** training loads, monitoring, illness, recovery, triathlon

## Abstract

Little is known about how recreational triathletes prepare for an Olympic distance event. The aim of this study was to identify the training characteristics of recreational-level triathletes within the competition period and assess how their preparation for a triathlon influences their health and their levels of fatigue. During the 6 weeks prior to, and the 2 weeks after, an Olympic distance triathlon, nine recreational athletes (five males, four females) completed a daily training log. Participants answered the Daily Analysis of Life Demands Questionnaire (DALDA), the Training Distress Scale (TDS) and the Alberta Swim Fatigue and Health Questionnaire weekly. The Recovery-Stress Questionnaire (REST-Q) was completed at the beginning of the study, on the day before the competition, and at the end of week 8. Training loads were calculated using session-based rating of perceived exertion (sRPE). The data from every week of training was compared to week 1 to determine how athletes’ training and health changed throughout the study. No changes in training loads, duration or training intensity distribution were seen in the weeks leading up to the competition. Training duration was significantly reduced in week 6 (*p* = 0.041, d = 1.58, 95% CI = 6.9, 421.9), while the number of sessions was reduced in week 6 (Z = 2.32, *p* = 0.02, ES = 0.88) and week 7 (Z = 2.31, *p* = 0.02, ES = 0.87). Training was characterized by large weekly variations in training loads and a high training intensity. No significant changes were seen in the DALDA, TDS or REST-Q questionnaire scores throughout the 8 weeks. Despite large spikes in training load and a high overall training intensity, these recreational-level triathletes were able to maintain their health in the 6 weeks of training prior to an Olympic distance triathlon.

## 1. Introduction

Triathlon is a unique sport that requires athletes to excel in swimming, cycling and running over a variety of distances. Amateur triathletes make up the majority of participants [1]. Success in the sport requires that triathletes possess above average aerobic power and muscular endurance, along with well-developed anaerobic capacities for surges in pace and for the final moments of the race [2,3,4]. To be able to prepare for the demands of the sport while mastering the three disciplines, and depending on race distance [2,5,6,7,8], age-group triathletes have been reported to train between 8 and 16 h per week.

To maintain this training volume, triathletes may continue to train even when injured, by increasing their training load in another exercise mode to that in which the injury was sustained [4,9,10]. This approach to management of training loads and shifting of training to other modes when injured may expose recreational level triathletes to higher levels of risk for sustaining negative, training related, health outcomes [11]. The intensity at which the training sessions are performed may also be an issue [12]. According to previous research, recreational endurance athletes often perform easy sessions at a pace that is considered too hard [13], whilst not pushing hard enough on the intense training days. This can lead to a program with a higher overall intensity. This itself is linked to delayed recovery following training [14], a greater potential for the occurrence of non-functional overreaching [2,13] and potentially a higher likelihood of the occurrence of injuries [4,15]. Too much intense training can also be detrimental to performance. In endurance sports, a polarized approach, with a focus on training at lower intensities (below the lactate threshold), and with few key sessions at higher intensities, is likely the most effective way to elicit performance improvements [16,17].

The training frequency that is involved in preparation for triathlon competition, and possible associated difficulty in balancing their training and other life commitments, may also lead recreational-level triathletes to experience high levels of stress [18]. General life stress can negatively influence athletes’ health status [19], blunt the adaptive response to endurance or resistance training programs [20,21] and moderate the relationship between fatigue and recovery [22]. The monitoring of amateur triathletes’ training loads and well-being, to help balance their training and life stress and to improve the chances of early detection of negative health and performance outcomes, is therefore important [23].

Training loads can be monitored with different methods that are related to changes in performance, health and fatigue [3,15,24]. The session rating of perceived exertion (sRPE) is accepted as a valid measure of training load. It may be predictive of illnesses when a spike in training load occurs [24]. Monitoring fatigue levels throughout a training program, however, can be a challenging task. Even though many physiological measures have been investigated, most show little validity or practical application [3,15]. In this context, subjective measures (such as questionnaire-based surveys of mood and perceived stress,) have proven as or more effective than objective measures (such as blood markers and heart rate responses) [15,23,25,26].

Even though far more recreational level age-groupers prepare for the (1.5 km swim, 40 km bike, 10 km run) Olympic distance (OD) competition than do so for the (3.9 km swim, 180 km bike, 42.2 km run) Ironman distance (IR) [1], little is known about their training practices and associated health status. Only one detailed longitudinal prospective investigation of training, maladaptation and training status markers in OD athletes appears to exist [27]. Vleck monitored 51 British National Squad OD specialists over a seven month lead up to the World OD Championships (weeks 20–23 of which included their World Championship qualifying and National Championship OD races). As her 1996 study predated the inception of draft legal racing for Elites, Vleck’s subjects were preparing for competition over what is nowadays the amateur OD triathlon format. However, their OD performance times over the study period (of 1:57:04 ± 0:19:55 hh:mm:ss for males and 2:10:30 ± 0:16:41 hh:mm:ss for females), would, even now, place them at the top level of such competition As such, they would be categorized as “well trained.” We are only aware of one (6 month) prospective longitudinal investigation of training volume and intensity distribution, in the lead up to competition, of recreational level triathletes [2]. The study of Neal et al. [2], however, involved IR distance athletes. The only, albeit retrospective, comparison of training in IR vs. OD athletes that exists [4] and that used the same methods of data collection for both groups, suggests them to differ in training practice. To date, no prospective longitudinal study of training and maladaptation in recreational level OD triathletes has been published in the academic literature.

The purpose of this study, therefore, was to monitor the training characteristics of recreational-level triathletes in the lead up to an OD triathlon competition and assess how the participants’ training influenced measures of health and fatigue. As the phenomenon has been reported with other endurance sports athletes [13], it was hypothesized that recreational triathletes would spend a disproportionate amount of their training time at higher exercise intensities. This would lead the participants to report high levels of fatigue, stress and negative health symptoms on a weekly basis.

## 2. Materials and Methods

The study was approved by a local Research Ethics Board (Pro00082267). Participants were informed of the risks and benefits of the study prior to signing an informed consent document. Recruitment occurred online via social media and the website of the following events: World Triathlon Series (WTS) Edmonton, WTS Montreal and the Vancouver Triathlon. All the events occurred between July and September of 2018.

### 2.1. Subjects

The participants were required to have 3 or more years of experience training for and competing in Olympic distance events. In this case, 11 participants (6 males, 5 females), with ages varying between 30 and 47 years old (39.2 ± 5.8, mean ± SD) volunteered for the study and competed in one of the aforementioned events. All the participants were classed as recreational athletes as they were competing in the amateur (“age-group”) category, were not part of a regional or national development center and trained and competed in their leisure time [7,8]. None reported training as their main occupation. The Olympic distance race times of our subjects were slower than those of well-trained male age-groupers who had similar triathlon training experience [28]. This confirms the recreational nature of the participants in the current study.

### 2.2. Measurements

The participants agreed to record their training programs within the 6 weeks leading up to an Olympic distance triathlon that was the key event of their season- and over the 2 weeks that followed the event. A questionnaire was used to cover participants’ experience in the sport (i.e., their years of training and competition), and how long they had been training or competing in swimming, cycling and running. Information on the participants’ best performance in prior Olympic distance events, the age at which they started training and competing in triathlon, and their past training practice (e.g., their hours of training per week, training frequency and longest session in each exercise mode) was also collected. Lastly, the participants were asked about how many triathlons (over any distance) they had competed in within the current and past years.

#### 2.2.1. Training Monitoring

Training was monitored via a customized online training log that was developed for this study. The participants were instructed to maintain their regular training programs while tracking every session. The training log required participants to report their session goal, activity type (e.g., tempo run, intervals), exercise mode and session rating of perceived exertion (sRPE) [24]. The study participants were also asked to report on other types of sessions that they performed (other modes of endurance training, such as rowing or resistance training sessions, for example) and describe what they were.

#### 2.2.2. Training Load Calculations

External training loads were calculated as the total duration of each session (in minutes) across each week, and separated by mode of training (swimming, cycling, running). The participants’ internal loads were calculated using the session rating of perceived exertion method (sRPE) that was developed by Foster [24], with the duration of each session multiplied by the rate of perceived exertion (1–10) that was assigned by the athlete to that session. Training monotony, an index of training variability defined as the daily mean load divided by the standard deviation of the load calculated over a week, was determined for each week [24]. Training strain (the product of training load and monotony) was also calculated weekly [24].

#### 2.2.3. Training Intensity Distribution (TID)

The TID of the athletes was calculated based on the sRPE that was reported for each session. This method was chosen as the researchers did not have data on the participants’ maximal heart rates, and it allowed for an easier collection of the intensity of the swimming sessions. Nevertheless, there is evidence to support the use of sRPE for assessing training intensity distribution in endurance athletes [16]. Sessions with a RPE of 4 or lower were considered as zone 1, a RPE of 5 or 6 were considered to equate to zone 2 and a RPE of 7 and above was considered as zone 3 [16]. The duration of each session was then assigned to its respective intensity zone (1, 2 or 3) so that the total amount of time within each zone could be calculated for each mode of exercise (swimming, cycling and running).

#### 2.2.4. Self-Reported Measures of Health, Fatigue and Illness

At the end of each training week, participants were sent three questionnaires: the Daily Analysis of Life Demands (DALDA), the Training Distress Questionnaire and the Alberta Swim Fatigue and Health Questionnaire (details of which are provided below), via a digital link. The athletes were instructed to return the completed forms to the researchers within 24 h of receipt.

The DALDA was formulated on the basis of multiple tests for response consistency that were conducted in swimmers [26]. The reliability criterion was a stress or source item being responded to in the exact same manner on four of five occasions, each 14 days apart, by at least 80% of athletes. If this was not the case, the item was deleted. After this initial evaluation, 9 questions to assess general stress levels and their source (part A) and another 25 questions aimed at determining symptoms of health and fatigue (part B), remained in the questionnaire. Completion of the DALDA involves the athlete rating himself/herself as being either “worse than normal”, “normal” or “better than normal” on each variable. As they can be representative of an increased level of (negative) stress [5,26], changes in the numbers of “worse than normal” scores are used to monitor athlete’s health. The DALDA can be repeatedly administered during a training period [26] and is sensitive to changes in training loads. In triathletes undergoing a period of intensified training, the questionnaire was considered a practical test to monitor changes in recovery and health [5].

The Training Distress Scale Questionnaire (TDS) [25] both quantifies the psychobiological response to training and helps identify athletes who are at risk of training-induced distress. The TDS was developed from the seven items of the Profile of Mood States (POMS) [29] that Raglin and Morgan [25] considered to be the best identifiers of an athlete in distress. The questionnaire was initially validated in swimmers, across seven time points of their season. It was found to have a mean successful prediction rate of 69.1% (*p* < 0.05). The scale was later both cross-validated and found to be equally effective at identifying athletes in distress, in track and field athletes [25]. The TDS consists of 7 items to which participants rate their mood responses using a 5-point Likert scale (that ranges from “0—not at all” to “4—extremely”). Lower overall scores are taken to mean that the athlete is displaying a better mood state.

Lastly, the Alberta Swim Fatigue and Health questionnaire (ASFH) [30] was used to determine health and fatigue status, as well as general attributes that are associated with good health, on a weekly basis. The ASFH has previously been used to detect changes in respiratory symptoms with manipulations in training load [30], whilst also being used to assess changes in health and fatigue in varsity athletes. The questionnaire collects information about the athletes’ overall wellbeing and health, in addition to their respiratory symptoms. Aches and soreness were identified as either a headache or general body ache (that was not specific), joint ache or pain, or muscle soreness, which was separated by body segments (e.g., lower back, shoulders, quadriceps, calves, etc.). A niggle was defined as a nagging pain that still allowed participants to train, although it could force participants to modify their training. If participants had to modify their training, the extent to which it was modified was also reported (i.e., no modification, to a minor extent, to a moderate extent, to a major extent, cannot participate at all).

At study baseline, 48 h prior to the OD race and 2 weeks post-event, the athletes also completed the Recovery Stress Questionnaire for Athletes (REST-Q). The REST-Q, which was initially validated in rowers [31], measures both the frequency of current stress and the frequency of recovery associated activities. It consists of 77 items (19 scales with four items each, plus a warm-up item), each with values ranging from 0 (never) to 6 (always), indicating how often the athlete has participated in activities over the past 3 days and nights [31]. Each of the scales has been rated as both reliable (with Cronbach’s α for each scale ranges of between 0.68 and 0.89), and to possess good test-retest reliability [31]. For the purposes of this study, total stress was calculated as the sum of the 10 stress subscales, while total recovery was calculated as the sum of the 9 recovery subscales. The athletes’ recovery-stress balance was calculated as the total stress score minus the total recovery score [6]. High scores in stress-associated scales reflect intense subjective strain, while high scores in the recovery associated scales reflect adequate recovery [6,31].

Saw et al. [23] and others [3,5,6,30] have demonstrated that such questionnaires can accurately reflect acute and chronic changes in well-being in response to alterations in training loads and are likely associated with changes in objective measures of training, health and fatigue. Previous studies in well-trained, but not elite, triathletes have also identified that questionnaires such as the DALDA and the REST-Q might play an important role in detecting alterations in levels of fatigue during a period of intensified training [5,6].

### 2.3. Data Analysis

Statistical analysis was performed using IBM SPSS^®^ Statistics v.24 (IBM, Armonk, New York, NY, USA), with the significance level set at *p* ≤ 0.05. Data distribution was checked with the Shapiro-Wilk test. A repeated-measures analysis of variance (ANOVA) was used to compare changes between week 1 and every subsequent week to analyze how training changed over time relative to baseline. Health and fatigue symptoms during the 8 weeks of the study were also compared to week 1. Partial eta square effect sizes are reported (η^2^) and interpreted as small (0.01), medium (0.06) and large (0.14). When a main effect of time was found, post-hoc comparisons were performed using the Bonferroni correction, with Cohen’s d calculated to report effect sizes (d) of pairwise comparisons between weeks (0–0.2 = trivial, 0.2–0.6 = small, 0.6–1.2 = moderate, 1.2–2.0 = large and >2 = very large) [32]. To control for alpha level inflation, only pairwise comparisons between week 1 and every subsequent week were performed. When Mauchly’s test was significant, a Greenhouse-Geiser adjustment was used to determine the significance level of the test. If the assumption of normality was violated, Friedman’s Test was utilized to assess the main effect of time, with Kendall’s W used to report effect sizes (*W*). When the main effect of time was significant, the Wilcoxon-Signed Rank test was used to determine differences between weeks, with effect sizes (ES) calculated for each comparison (r = Z/√N) and interpreted as 0.10–small, 0.30–moderate, and 0.50–large effect [33]. Correlation analyses between weekly training characteristics (training loads, monotony and strain, and training time in zones 2 and 3) and both the “worse than normal” scores on the DALDA questionnaire and the scores on the TDS were performed using Spearman’s Rank Test.

## 3. Results

The data of two individuals (one male and one female) were excluded from analysis, as a result of failure to complete the training log or to maintain a training routine. The final study subject number was nine, therefore. The characteristics of the study participants are presented in Table 1.

Two athletes won their age-group category, and one did not finish the race due to injury. Given their age and/or the number of finishers in each of their respective age-groups, we were able to confirm that the athletes in our study would not be classified as “fast, well-trained age-group Olympic distance triathlete” (s) [28]. Rather, this study deals with recreational level age-group triathletes. According to the training history questionnaire, the participants had an average of 5 years of experience in triathlon and a greater training history in one of the disciplines, with running being the most common. The athletes reported performing more cycling and running sessions in a week than swimming sessions. The number of cycling and running sessions that were accomplished in a week were similar.

### 3.1. Training Characteristics

The training characteristics of the participants is presented in Table 2.

#### 3.1.1. Training Duration (Min) and Time Spent within Each Training Zone

Total training duration changed significantly over the 8 weeks (F(7, 56) = 4.126, *p* = 0.014, η^2^ = 0.340). When compared to week 1, total training volume was significantly lower in week 6 (*p* = 0.041, 95% CI = 6.93, 421.95, d =1.58, 414.08 ± 170.5 vs. 199.6 ± 97.6 min). Over the 8 weeks of training, no significant changes were seen in the overall time spent in zone 1 (F(7, 56) = 1.225, *p* = 0.305, η^2^ = 0.133) or zone 2 (χ^2^ (7) = 9.00, *p* = 0.252, W = 0.143). However, a significant difference for time spent in zone 3 was found (χ^2^ (7) = 22.56, *p* = 0.002, W = 0.358). Compared to week 1, the time spent in zone 3 was significantly shorter in week 6 (Z = 2.52, *p* = 0.012, ES = 0.95, median = 78 vs. 0 min), week 7 (Z = 2.10, *p* = 0.036, ES = 0.79, median = 78 vs. 0 min) and week 8 (Z = 1.960, *p* = 0.050, ES = 0.74, median = 78 vs. 40 min).

#### 3.1.2. Total Time Spent Swimming, Cycling and Running and Time Spent in Zones 1, 2 and 3 for Each Mode

No significant differences across the 8 weeks were found for total swimming time (χ^2^ (7) = 10.29, *p* = 0.173, W = 0.163). Total cycling time (F(7, 56) = 2.483, *p* = 0.027, η^2^ = 0.237) was significantly changed across the 8 weeks, but no differences in relation to week 1 were found. Total running time differed across the 8 weeks (χ^2^ (7) = 16.39, *p* = 0.022, W = 0.260), with running time in week 6 (Z = 2.54, *p* = 0.011, ES = 0.96, median = 27 vs. 105 min) and week 7 (Z = 2.07, *p* = 0.038, ES = 0.78, median = 31 vs. 105 min) being lower when compared to week 1. No differences were found for time spent in zones 1 or 2 for swimming, cycling or running. Time spent in zone 3 was significantly different across weeks for swimming (χ^2^ (7) = 16.21, *p* = 0.023, W = 0.257) and cycling (χ^2^ (7) = 23.33, *p* = 0.001, W = 0.370). However, post-hoc comparison only showed a significant difference between week 1 and week 6 for cycling in zone 3 (Z = 2.023, *p* = 0.043, ES = 0.76, median = 25 vs. 0 min). For running, time spent in zone 3 was significantly different across the 8 weeks (χ^2^ (7) = 19.52, *p* = 0.007, W = 0.310), with week 6 (Z = 2.36, *p* = 0.018, ES = 0.89, median = 0 vs. 53 min) and week 7 (Z = 2.36, *p* = 0.018, ES = 0.89, median = 0 vs. 53 min) presenting a significantly lower duration at this intensity compared to week 1.

#### 3.1.3. Number of Sessions per Week

There was a significant difference in the number of sessions performed each week (χ^2^ (7) = 19.04, *p* = 0.007, W = 0.308). When compared to week 1, participants maintained their training frequency until week 6, when frequency was reduced (Z = 2.32, *p* = 0.02, ES = 0.88, median = 6.0 vs. 9.0). Training frequency was also reduced the week after the competition, with week 7 being significantly different than week 1 (Z = 2.31, *p* = 0.02, ES = 0.87, median = 5.0 vs. 9.0).

There was no difference in the number of swimming (χ^2^ (7) = 5.88, *p* = 0.553, W = 0.09) and cycling sessions (χ^2^ (7) = 10.16, *p* = 0.180, W = 0.16) that were performed over the 8 weeks. However, the number of running sessions changed significantly (χ^2^ (7) = 16.82, *p* = 0.019, W = 0.26), with a higher number of sessions on week 1 when compared to week 7 (Z = 1.98, *p* = 0.048, ES = 0.75, median = 3.0 vs. 1.0 sessions). The number of other types of sessions performed throughout the 8 weeks did not change (χ^2^ (7) = 10.92, *p* = 0.142, W = 0.17). Of the other sessions performed, only 1 participant performed some form of cross training, with the rowing and paddling sessions included in the training load calculations. Resistance training and yoga were the only other types of session that were performed and were not included in the training load calculations.

### 3.2. Training Load, Training Monotony and Training Strain

Whilst training loads changed significantly over time (F(7, 56) = 3.971, *p* = 0.001, η^2^ = 0.332), no differences were found between week 1 and the other weeks of training. The average training load of the event was 1371.2 ± 248.2 A.U. To assess if the overall load was reduced in the week of the event, the training load for week 6 was calculated with and without the load from the event. Removing the competition load from the training load calculations for week 6 did not lead to a significant difference between week 1 and week 6. Training monotony changed significantly during the 8 weeks (χ^2^ (7) = 19.07, *p* = 0.008, W = 0.30), with a significantly lower value for week 6 when compared to week 1 (Z = 2.54, *p* = 0.011, ES = 0.96, median = 1.0 vs. 0.6). Similarly, significant differences over the 8 weeks were reported for training strain (χ^2^ (7) = 16.11, *p* = 0.024, W = 0.25), with pairwise comparisons showing a higher training strain during week 1 when compared to week 6 (Z = 2.42, *p* = 0.015, ES = 0.91, median = 2322.1 vs. 1403.8).

### 3.3. Self-Reported Measures of Health, Fatigue and Stress

No significant changes were reported for the DALDA questionnaire (χ^2^ (7) = 12.54, *p* = 0.084, W = 0.224) and the Training Distress Scale (χ^2^ (7) = 9.01, *p* = 0.252, W = 0.16) throughout the 8 weeks. For the REST-Q, no significant differences were found among responses at baseline, 48 h prior to the event, or two weeks after it (F(2, 14) = 0.803, *p* = 0.46, η^2^ = 0.103).

For the Alberta Swim Fatigue and Health Questionnaire, weeks 6 and 7 presented some of the lowest reports of negative health symptoms (i.e., cold, flu, upset stomach, not feeling good overall), muscular aches and soreness and niggles. Symptoms were reported every week by at least 40% of participants, and every week at least 2 participants reported that they had to modify their training (Table 3).

#### Correlation Analysis between Training Loads and Questionnaire Responses

No significant correlations were observed between training loads, monotony or strain and participants’ responses to the DALDA or Training Distress questionnaire. Similarly, no significant correlation was found between the time spent in either zone 2 or zone 3 and the scores on each questionnaire.

## 4. Discussion

This study examined the training characteristics of recreational-level triathletes in the 6 weeks leading up to an Olympic distance triathlon and the 2 weeks after the event. The participants in this study had a training frequency that ranged between 5 and 9 sessions per week. The weekly training duration averaged 6.2 h per week from weeks 1 to 5, with weeks 7 and 8 showing a decrease in training duration. Not considering the athletes’ Olympic distance triathlon, week 6 saw a significant reduction in training duration when compared to week 1, with athletes averaging just under 3 h of training. These training volumes are below what has been reported for 16 well-trained, but not elite triathletes, who had a minimum weekly training volume of 10 h [6]. Compared to athletes training for longer distance triathlons, the average weekly training volume was also lower, as previous research has identified that recreational-level Ironman triathletes train on average 14.1 h per week [7,8]. While these differences can be expected given the duration of the events (Ironman vs. Olympic distance), it must be acknowledged that the difference can be in part explained by the fact that data collected prospectively, such as in this study, can differ from retrospective data, as in the above-mentioned study. Nevertheless, training volume in this group of recreational triathletes was still larger than single mode recreational endurance athletes, such as half-marathon and marathon runners [7,34], and cyclists with similar years of experience as the athletes in this study [35].

**Figure 1:**
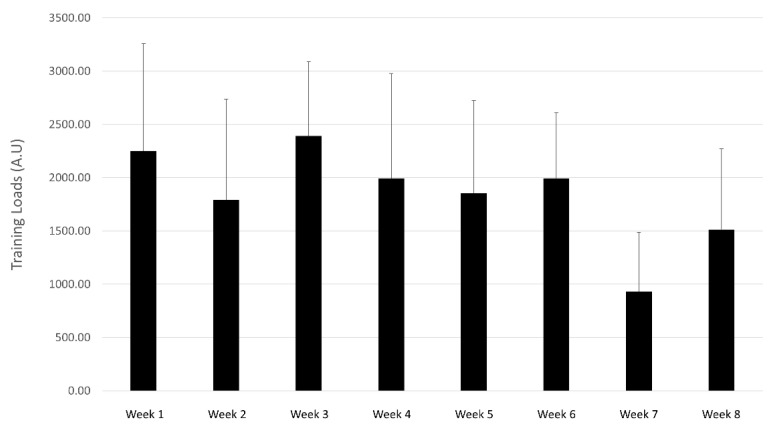
Average training loads (A.U) throughout the 8 weeks of training.

Despite the importance of monitoring and reporting training volume, training loads are more relevant as these can determine if an athlete is adapting to the training program, assess fatigue and recovery status, and minimize the risks of non-functional overreaching, injury and illness [15]. The average load (Figure 1) in the weeks prior to the competition (2150.01 A. U., from weeks 1 to 5) is slightly higher than the average of 2000 A.U [6] reported by a group of well-trained male triathletes completing a four-week progressive, self-prescribed loading regime. While similar, the higher loads in the present study were achieved despite a lower average weekly training volume, indicating that weekly sessions were perceived to be performed at a higher intensity in this group of athletes. In addition, some of the reported loads in the current study were surprisingly high. For example, Coutts et al. [6] put a group of participants through a 4-week period of training overload designed to lead to overreaching. Weekly training loads started at upwards of 3000 A.U, a value that was reached by 5 of the 9 participants in this study at least once during the 8 weeks. One participant in this study also had a weekly load greater than what was reported by Coutts et al. [6] during their second week of overload (3.884 A.U vs. 3.809 A.U).

The training loads of the participants in this study were characterized by a high degree of variability throughout the eight weeks, with no discernible pattern in the five weeks leading up to the event. For the whole group, loads were reduced by 17% from week 1 to week 2 (2292.4 vs. 1911.2 A.U), only to increase by 27% (1911.2 vs. 2429.7) in week 3. While loads were reduced by an average of 10% in weeks 4 and 5 (Figure 1), an analysis of individual numbers confirms that large spikes in loads between weeks were frequent (Supplementary Material). Indeed, all participants doubled their loads from the previous week at least once throughout the study. As an example, one participant had a reduction in load of 33% in week 3 compared to week 2 (1064.2 A.U and 1603.4 A.U, respectively), followed by an increase of 122% in week 4 (2362.3 A.U), and a reduction of 60% in week 5 (948 A.U). These large variations in training loads can be detrimental to athletes’ health. An association between training loads and injuries has been established, with large spikes in loads linked to an increased chance of injury, with the risk potentially remaining elevated for many weeks [36]. These spikes in load could also be related to an increased incidence of banal infections, a potential early sign of non-functional overreaching [24].

**Figure 2:**
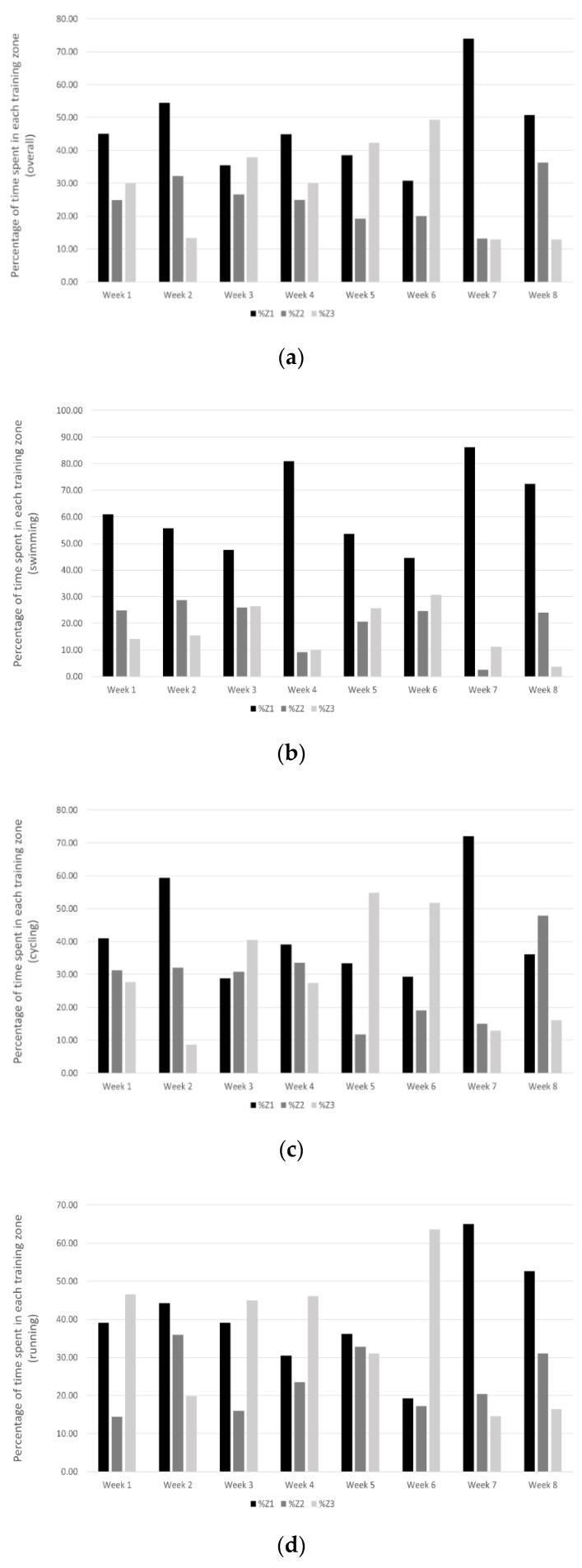
Training intensity distribution based on 3-zone model across the 8 weeks of training. (**a**) overall, (**b**) swimming, (**c**) cycling, (**d**) running. %Z1 = percent of time spent in zone 1, %Z2 = percent of time spent in zone 2, %Z3 = percent of time spent in zone 3.

Similar to what occurred with training loads, no pattern was seen in the changes in training intensity distribution throughout the weeks of training (Figure 2). A high variability in the percentage of time spent in each training zone throughout the training program was also found. The only difference in TID found throughout the study was in the amount of training that was performed in Zone 3. Due to the competition, the athletes spent a greater amount of time in this intensity zone during week 6, and subsequently reduced the amount of time training in this zone in the two weeks after the competition. In addition, the athletes’ training intensity over the 8 weeks confirmed our initial hypothesis, with participants’ training intensity distribution favoring higher intensity sessions. Particularly, athletes spent an average of 47% of their training time in zone 1, with more than half of training spent in zones 2 and 3 (25% and 28%, respectively). When the time spent in zones 2 and 3 is considered together, only 2 out of the 8 weeks had a greater amount of time in zone 1 than in zones 2 and 3.

The athletes’ TID varied for each discipline, with swimming having a higher percentage of training time in zone 1 when compared to cycling and running. As many overuse injuries in triathlon are associated with cycling and running, particularly with the performance of intense sessions [4], the high volume of training in zones 2 and 3 in these disciplines could be cause for concern. For example, over the 8 weeks of training, the time spent in zone 3 during cycling was higher than that in zone 1 in weeks 3 and 5. Similar results were seen in the TID in running, where the amount of time spent in zone 3 was higher than that in zone 1 in 3 of the 5 weeks prior to the competition.

This high volume of training spent at higher intensities can be detrimental to athletes’ performance. Improvements in endurance performance have been shown to be inversely related to the time spent at threshold intensities [2,17], with previous research showing that adaptations to training are related to the time spent in zone 1 (below the first ventilatory threshold) [37]. In addition, too much training time in zone 2 (between the first and second ventilatory thresholds) is also linked to symptoms of non-functional overreaching and a higher incidence of injuries [3,4,13]. Furthermore, Seiler et al. [14] demonstrated that training above the first ventilatory threshold (VT1), which demarcates the upper range of zone 1, can significantly delay recovery. This is particularly troubling for sports where training frequency can be high, such as triathlon, since it is possible that athletes would not be fully recovered prior to the following session.

Nevertheless, despite the large spikes in training loads between weeks and a training intensity distribution that favored higher intensities, contrary to our hypothesis, there were no significant changes in the athletes’ fatigue and recovery status based on the questionnaires that were used. Only two injuries were reported, with one being due to a fall from the bike. However, high scores in the questionnaires, indicating a lack of recovery or presence of negative health symptoms were seen even in weeks with lower training loads. As recreational athletes struggle to maintain a balance between training and their regular life commitments [18], it is possible that general stress has an even greater impact on these athletes’ self-reported measures of fatigue, illness and health. Otter et al. [22] reported that in a group of female endurance athletes (including five triathletes), recovery was hindered throughout the year of training in moments when general stress was higher. Further evidence also exists to support the notion that amateur triathletes have more difficulty in dealing with stress than those at the elite level [38], and other studies [20,21] have shown that for the general population, a cautious approach would be advisable when engaging in strenuous exercise if under chronic stress.

Even though these athletes were apparently healthy according to standard measures of fatigue, recovery and health (the DALDA, the TDS and the REST-Q), signs and symptoms associated with excessive training and not enough recovery were evident. Particularly, muscle soreness, aches and niggles were reported in the Alberta Swim Fatigue and Health Questionnaire every week by at least 40% of the athletes, with 20% of them having to modify their training on a weekly basis. This modification to training is similar to what has been previously reported in the literature, with athletes often increasing the load in another discipline when necessary [3]. While further research is needed to understand these athletes’ approach to training, it is possible that the need to modify their training and the participants’ approach to managing their complaints of muscle soreness, niggles and aches could help in explaining the high variations in weekly training loads.

In summary, the results of this study show that the participants incurred no significant changes in their health status in the 6 weeks prior to, or within two weeks after, competing in an OD triathlon. This situation occurred in spite of the athletes not having appeared to follow what is currently considered to be the ideal training practice for training for endurance events. Certainly, recreational triathletes should attempt to minimize large variations in training loads (as were reported for this group)-as such variations may have negative consequences for health and performance [2,6,24]. Age-group triathletes may also benefit from performing a higher proportion of their training at lower intensities (i.e., zone 1 in a 3-zone model), whilst limiting the volume of training that is accomplished above the first ventilatory threshold. In this group [2], performance improvements have been associated with a higher training volume in zone 1 and a reduced volume of training in zone 2. Doing high volumes of intense training sessions may also augment the risk of the athlete sustaining overuse injuries [4,15]. We recognize that working with a coach might lead to a reduction, on the part of the athlete, in the incidence of such training errors. We acknowledge, however, that such errors may persist even when the athlete’s sessions are prescribed by a coach, if he/she performs the training at a different intensity than that which has been advocated [13].

Whilst this exploratory investigation has brought important information for coaches and athletes to light, the results should be interpreted with caution. For example, the small number of participants may partly explain why symptoms of fatigue were identified by the Alberta Swim Fatigue Health Questionnaire, but not by the other self-reported measures of health and fatigue that were implemented with them. Even though the nine athletes in question all exhibited similar training patterns (i.e., large variations in training loads and high volume of training being performed in intensity zones 2 and 3), the sample size of this study also precludes the drawing of definitive conclusions from it about recreational level triathletes as a whole. Previous research has also suggested that subjective measures of training maladaptation can be associated with, and, ideally, should be monitored alongside, objective measures [23]. Longer prospective longitudinal studies, that involve more participants; and that assess both subjective and objective measures of health, fatigue and performance, are needed if we are to better understand the inter-relationships between training characteristics and health in recreational triathletes.

## 5. Conclusions

The cohort of age-group triathletes in this study presented a random pattern of training throughout the 6 weeks prior to the competition, with large variations in training loads between weeks, along with several sessions performed at higher intensities (zones 2 and 3). Such approach to training could lead to a greater incidence of injuries, lack of recovery and reduced performance [2,4,14,24]. Nevertheless, no changes in the participants’ fatigue and recovery status were found with the DALDA and TDS questionnaires. Still, this information was captured by the Alberta Swim Fatigue and Health Questionnaire. It is possible that training for a competitive event for some of the recreational athletes in this group was a balancing act between the hours of training and general life. This corroborates a recent study in which recreational endurance athletes, particularly triathletes, reported their struggle to find the time to train [18] and how they felt the need to push beyond their comfort levels to stimulate the desired adaptations. While such behaviors could help explain the results seen in this study, further research should assess the training characteristics of recreational athletes and seek to both understand the reasons behind their training patterns, and how such training patterns impact the athletes’ health and performance.

## Figures and Tables

**Table 1:** Participants’ self-reported training characteristics.

Characteristics	Mean (Range)
Age when started triathlon training (years)	33.6 (19–42)
Experience of training and competing in triathlon (years)	4.5 (3–12)
Swimming Experience (years)	6.5 (3–15)
Cycling Experience (years)	7.0 (3–15)
Running Experience (years)	14.2 (3–30)
Number of triathlons performed last season (any distance)	3.7 (2–5)
Training volume in the previous year (hours) ^#^	341 ± 185 (150–674)
Number of triathlon specific sessions per week in the past year (overall and per mode) ^#^	7.3 ± 1.5 (5.0–9.0)
Swimming ^#^	2.2 ± 1.0 (1.0–4.0)
Cycling	3.0 (2.0–3.5)
Running ^#^	2.7 ± 0.6 (2.0–4.0)
Training volume per week in the past year (hh:mm:ss)	
Overall	08:48:00 (3:30:00–13:30:00)
Swimming	2:24:00 (00:30:00–05:00:00)
Cycling	03:48:00 (01:12:00–06:00:00)
Running	02:24:00 (01:00:00–06:00:00)
Longest session (hh:mm:ss) in the past year	
Swimming	01:16:36 (00:50:21–02:00:00)
Cycling	03:18:24 (01:30:00–07:00:00)
Running	01:52:20 (01:00:00–03:00:00)
Average finishing time for Olympic Distance triathlon in week 6 (range) (hh:mm:ss)	02:39:06 (02:25:00–02:51:45)
Males	2:36:00 (2:17:12–2:58:00)
Females	2:40:48 (2:30:30–2:46:00)

^#^ Data presented as mean ± standard deviation.

**Table 2:** Training characteristics of recreational-level triathletes for 8 weeks (data presented as median, 25th percentile, 75th percentile).

Variable	Week 1	Week 2	Week 3	Week 4	Week 5	Week 6	Week 7	Week 8	*p*-Value	Effect Size
Training Loads sRPE (A.U) ^#^	2292.4 ± 1058. 5	1911.2 ± 915.4	2429.7 ± 729.1	2166.7 ± 866.4	1949.9 ± 864.1	2129.6 ± 465.5	968.4 ± 577.3	1665.8 ± 621.3	<0.001	η^2^ = 0.447
Training Duration (hh:mm:ss)	07:43:48 (03:54:12, 09:05:05)	06:52:48 (04:40:12, 08:46:30)	06:49:00 (05:56:36, 08:42:42)	06:37:48 (03:28:12, 07:53:24)	04:54:00 (03:51:36, 07:59:24)	02:58:48 (02:01:12, 04:15:00) ^a^	02:39:36 (01:55:12, 06:04:12)	04:56:24 (02:03:36, 07:037:48)	0.014	η^2^ = 0.340
Time in Zone 1 (hh:mm:ss)	02:53:24 (01:45:00, 04:36:00)	02:55:12 (01:27:00, 05:06:00)	02:00:00 (00:00:00, 03:34:12)	02:45:36 (01:05:24, 04:27:00)	01:54:36 (00:34:00, 03:42:00)	02:00:00 (01:02:30, 02:34:48)	02:33:00 (00:45:12, 05:23:24)	02:18:00 (00:37:30, 04:39:00)	0.305	η^2^ = 0.133
Time in Zone 2 (hh:mm:ss)	00:39:00 (00:00:00, 02:24:06)	01:37:12 (00:37:18, 03:46:30)	02:03:00 (00:28:30, 03:00:06)	00:40:00 (00:00:00, 03:01:30)	01:00:00 (00:00:00, 02:09:00)	01:07:00 (00:00:00, 02:34:48)	00:00:00 (00:00:00, 00:43:42)	01:15:00 (00:38:30, 03:31:48)	0.252	*W* = 0.143
Time in Zone 3 (hh:mm:ss)	01:18:00 (00:46:00, 03:00:00)	00:28:00 (00:00:00, 01:42:00)	03:42:36 (00:55:00, 04:28:48)	01:36:00 (00:00:00, 03:24:00)	01:26:00 (00:35:30, 04:09:00)	00:00:00 (00:00:00, 00:55:52) ^a^	00:00:00 (00:00:00, 00:40:30) ^a^	00:40:00 (00:06:00, 01:09:00) ^a^	0.002	*W* = 0.358
Training Monotony (A.U)	1.0 (0.9, 1.3)	1.2 (0.8, 1.3)	0.7 (0.6, 1.5)	1.1 (0.8, 1.4)	0.9 (0.8, 1.4)	0.6 (0.6, 0.8) ^a^	0.7 (0.6, 1.3)	0.9 (0.8, 1.2)	0.008	*W =* 0.303
Training Strain (A.U)	2322.1 (1585.5, 2910.4)	2206.0 (1214.8, 3376.8)	1930.9 (1338.1, 3871.0)	2908.6 (1210.6, 3790.4)	1800.9 (1253.1, 3144.3)	1403.8 (1070.5, 1716.3) ^a^	601.1 (363.7, 1927.6)	1545.8 (1147.1, 2592.2)	0.024	*W =* 0.256
Number of Sessions per week	9.0 (5.5, 10.5)	7.0 (6.5, 8.0)	8.0 (6.0, 9.5)	7.0 (6.0, 9.0)	6.0 (5.0, 8.0)	6.0 (4.0, 6.5) ^a^	5.0 (4.0, 8.0) ^a^	7.0 (6.0, 8.5)	0.007	*W* = 0.308
Swimming sessions per week	2.0 (1.0, 3.0)	2.0 (1.0, 3.0)	3.0 (2.0, 3.0)	1.0 (1.0, 3.0)	2.0 (1.0, 3.0)	2.0 (1.0, 2.5)	1.0 (1.0, 2.5)	2.0 (0.0, 3.0)	0.553	*W* = 0.093
Cycling sessions per week	2.0 (1.5, 3.5)	3.0 (2.0, 3.0)	3.0 (2.0, 3.0)	2.0 (2.0, 3.0)	3.0 (1.0, 4.0)	2.0 (1.0, 3.0)	1.0 (1.0, 2.5)	1.0 (0.0, 3.5)	0.180	*W =* 0.161
Running sessions per week	3.0 (2.0, 3.5)	2.0 (1.5, 3.0)	2.0 (1.0, 3.0)	2.0 (0.0, 3.5)	2.0 (0.5, 3.0)	2.0 (0.5, 2.0)	1.0 (0,0, 2.0) ^a^	3.0 (2.0, 3.0)	0.019	*W* = 0.267
Number of other sessions per week	1.0 (0.0, 2.0)	0.0 (0.0, 1.0)	0.0 (0.0, 1.0)	1.0 (0.0, 1.0)	0.0 (0.0, 1.5)	0.0 (0.0, 0.5)	1.0 (0.0, 1.5)	0.0 (0.0, 3.0)	0.142	*W =* 0.173

^a^ denotes a significant difference from week 1 (*p* < 0.05); ^#^ data presented as mean ± standard deviation; A.U (arbitrary units).

**Table 3:** Descriptive athletes’ health status data according to the Alberta Swim Fatigue and Health Questionnaire (n = 9).

	Week 1	Week 2	Week 3	Week 4	Week 5	Week 6	Week 7	Week 8
Number of athletes who reported aches and soreness	6	7	5	9	7	5	9	6
Number of athletes who reported niggles	8	7	7	7	6	5	7	6
Number of athletes who modified training	3	5	2	5	3	3	2	3

## Data Availability

The data presented in this study are available on request from the corresponding author. The data are not publicly available due to privacy reasons.

## Supplementary Materials

The following are available online at https://www.mdpi.com/article/10.3390/sports9070094/s1, Figures S1–S9: Individual Training Loads for each participant.
