# Supplementary figures and images for: The Training Characteristics of Recreational-Level Triathletes: Influence on Fatigue and Health
Source: Sports (Basel). 2021 Jun 25;9(7):94. doi: 10.3390/sports9070094 (PMC8309729; doi:10.3390/sports9070094)

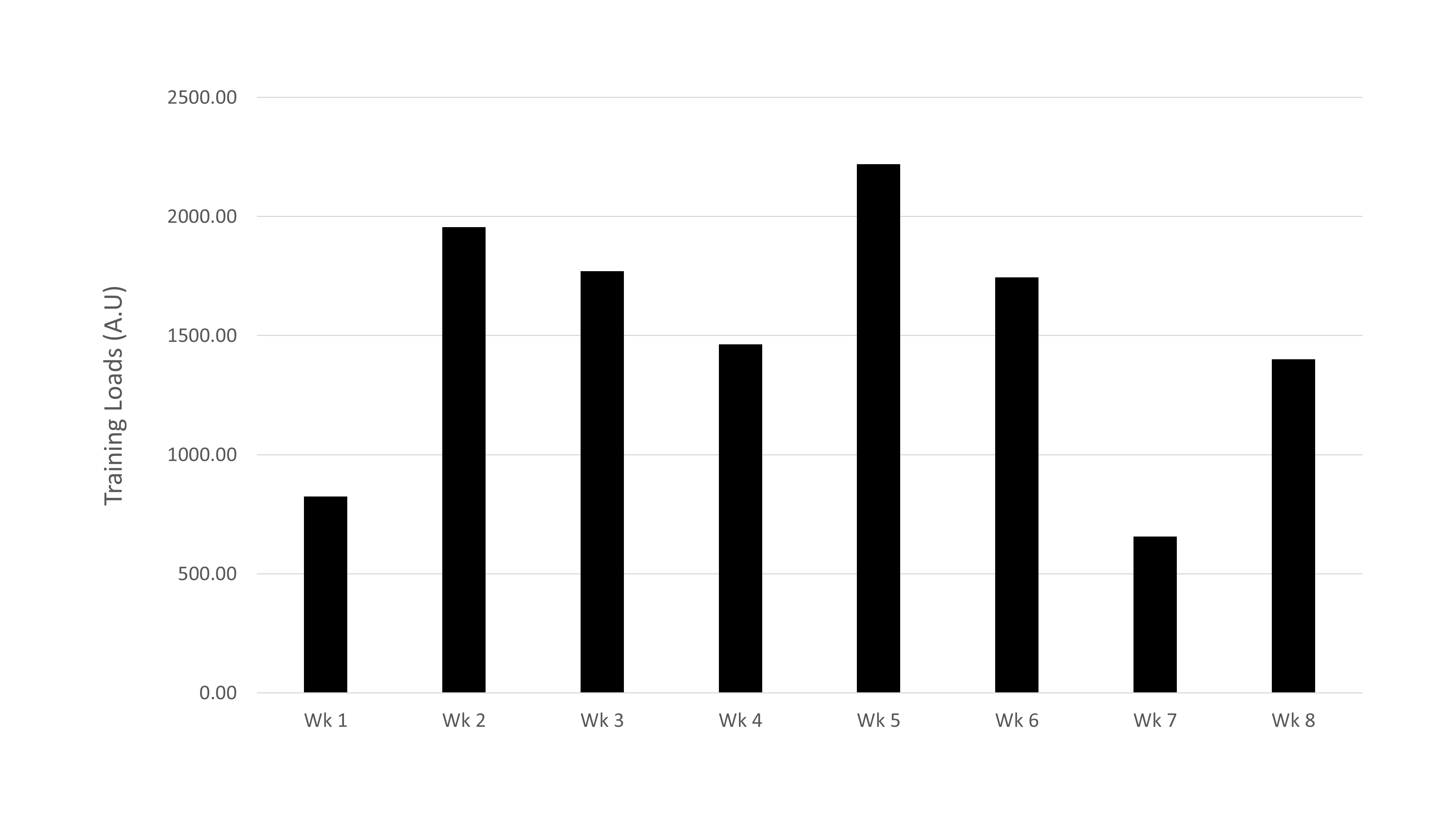

Supplement: Supplementary file 1 [file sports-09-00094-s001.zip › sports-1197701-SM/Figure S1 TL P1.tif]

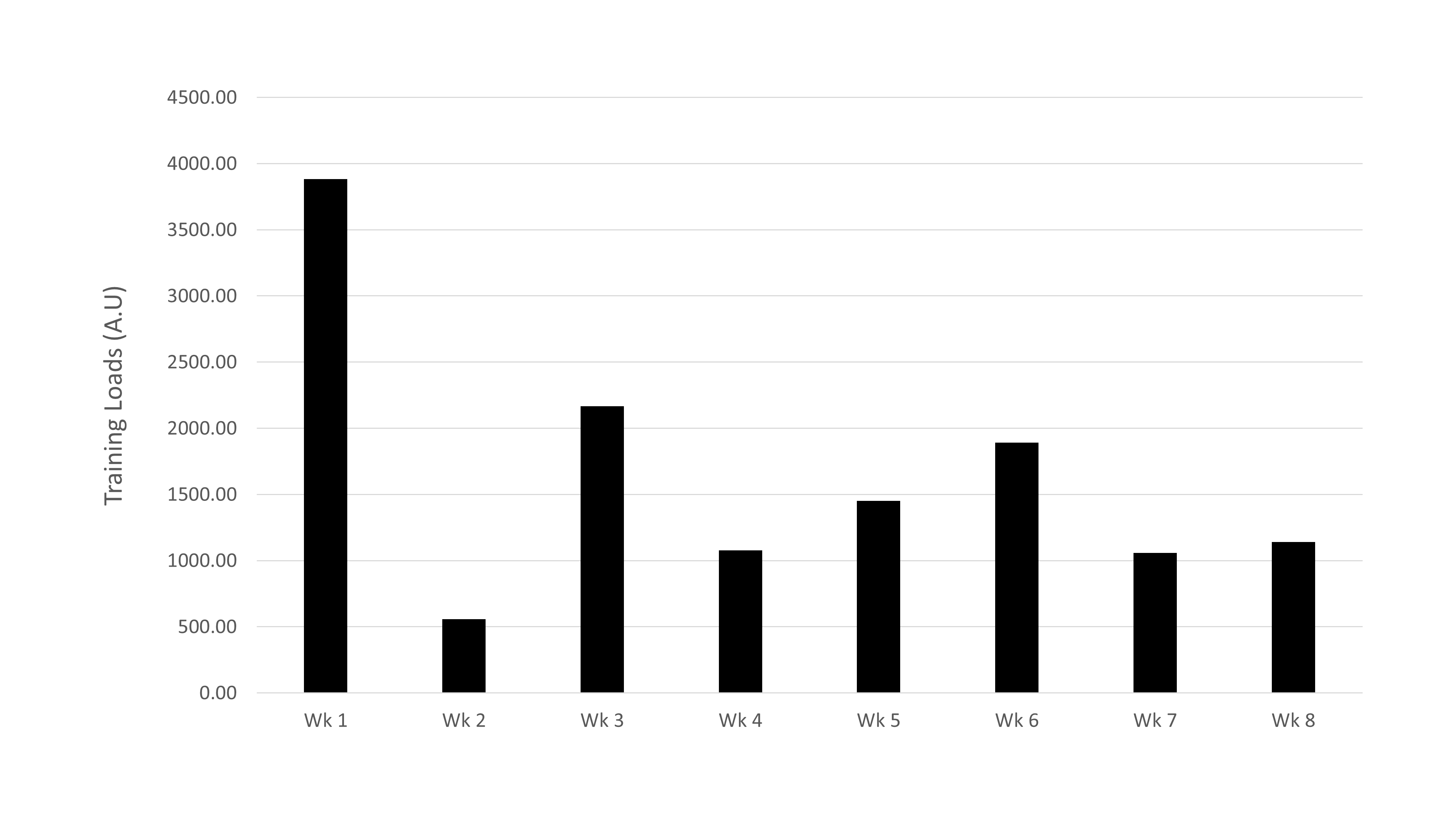

Supplement: Supplementary file 1 [file sports-09-00094-s001.zip › sports-1197701-SM/Figure S2 TL P2.tif]

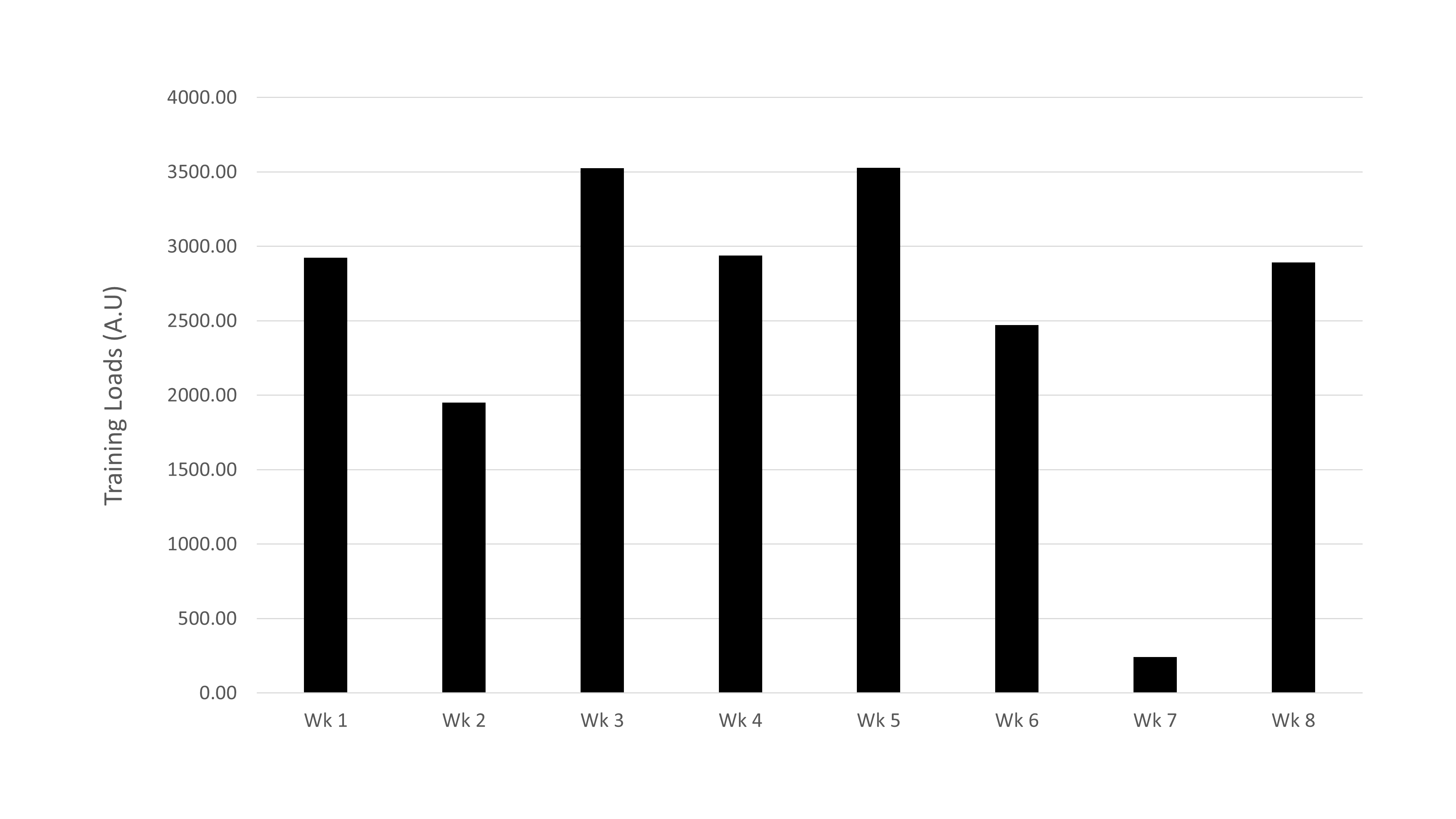

Supplement: Supplementary file 1 [file sports-09-00094-s001.zip › sports-1197701-SM/Figure S3 TL P3.tif]

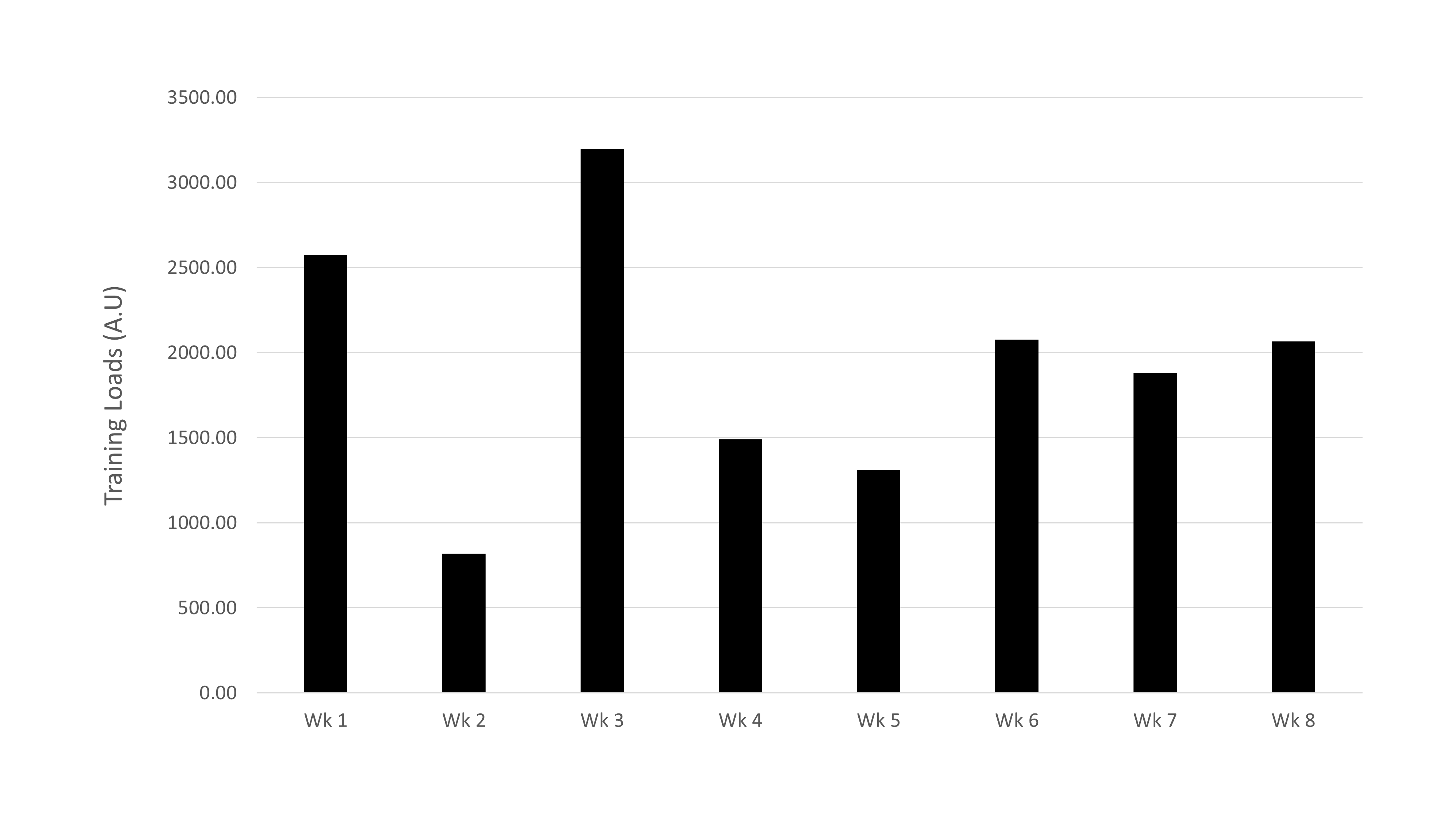

Supplement: Supplementary file 1 [file sports-09-00094-s001.zip › sports-1197701-SM/Figure S4 TL P4.tif]

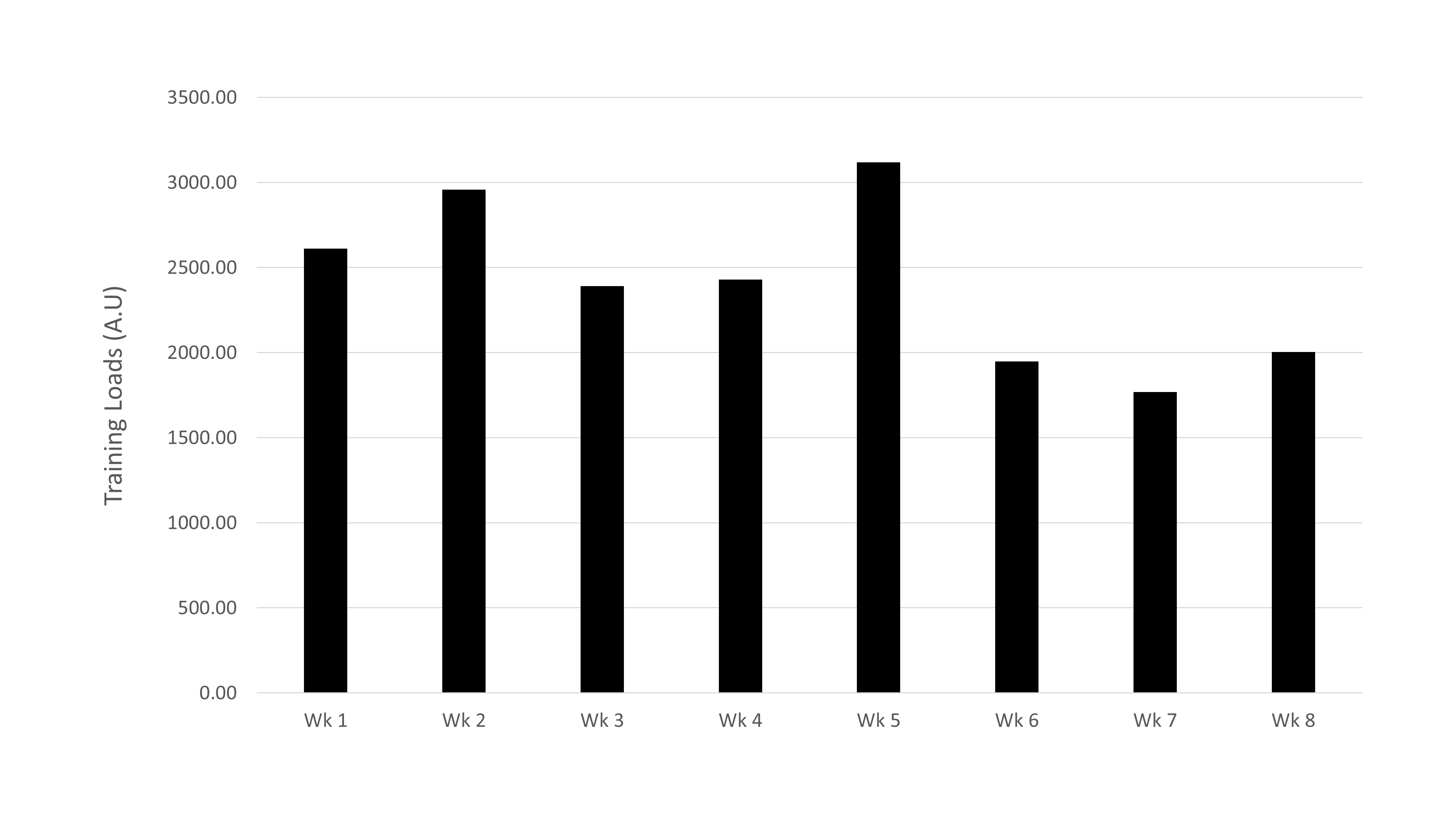

Supplement: Supplementary file 1 [file sports-09-00094-s001.zip › sports-1197701-SM/Figure S5 TL P5.tif]

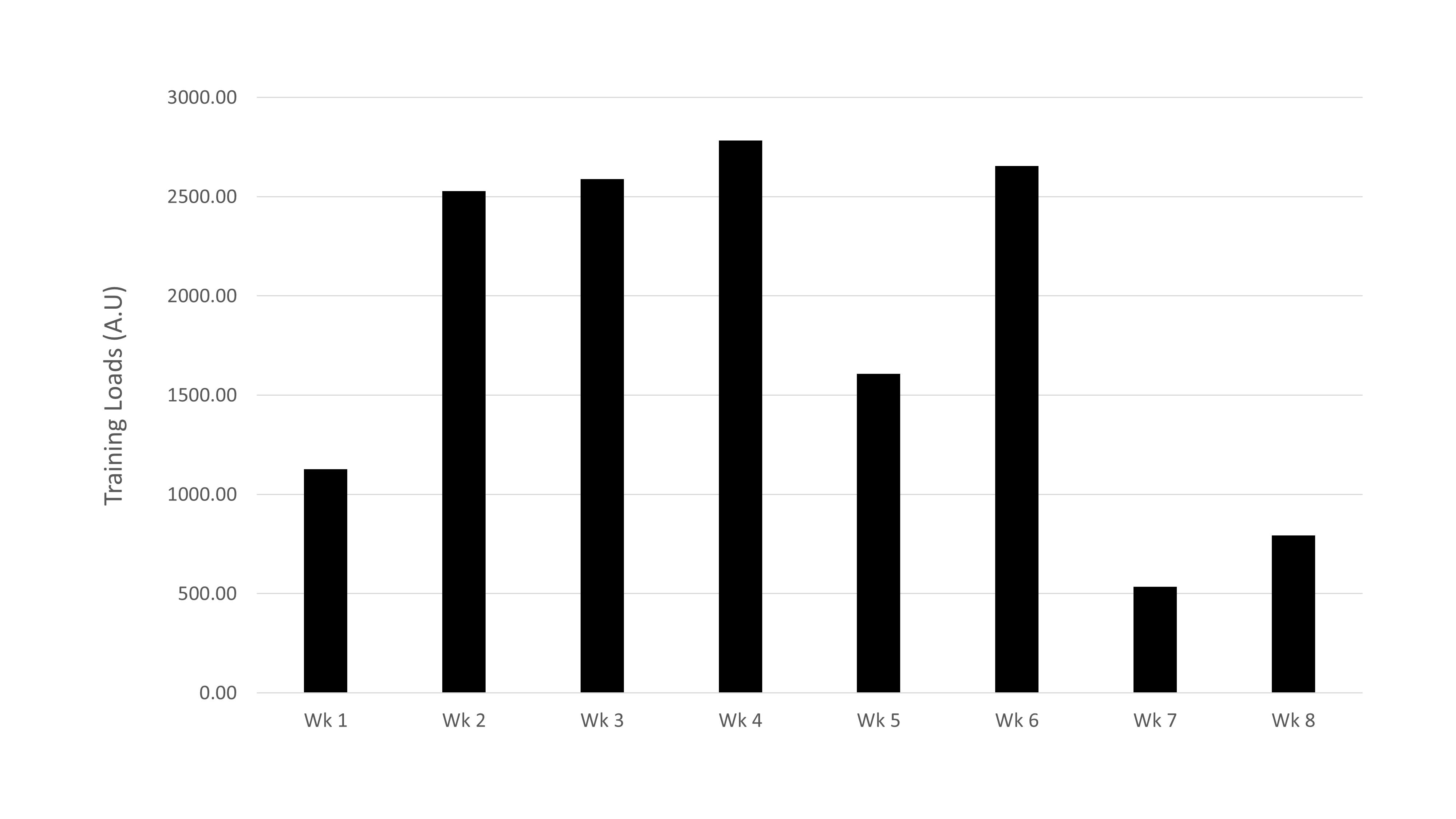

Supplement: Supplementary file 1 [file sports-09-00094-s001.zip › sports-1197701-SM/Figure S6 TL P6.tif]

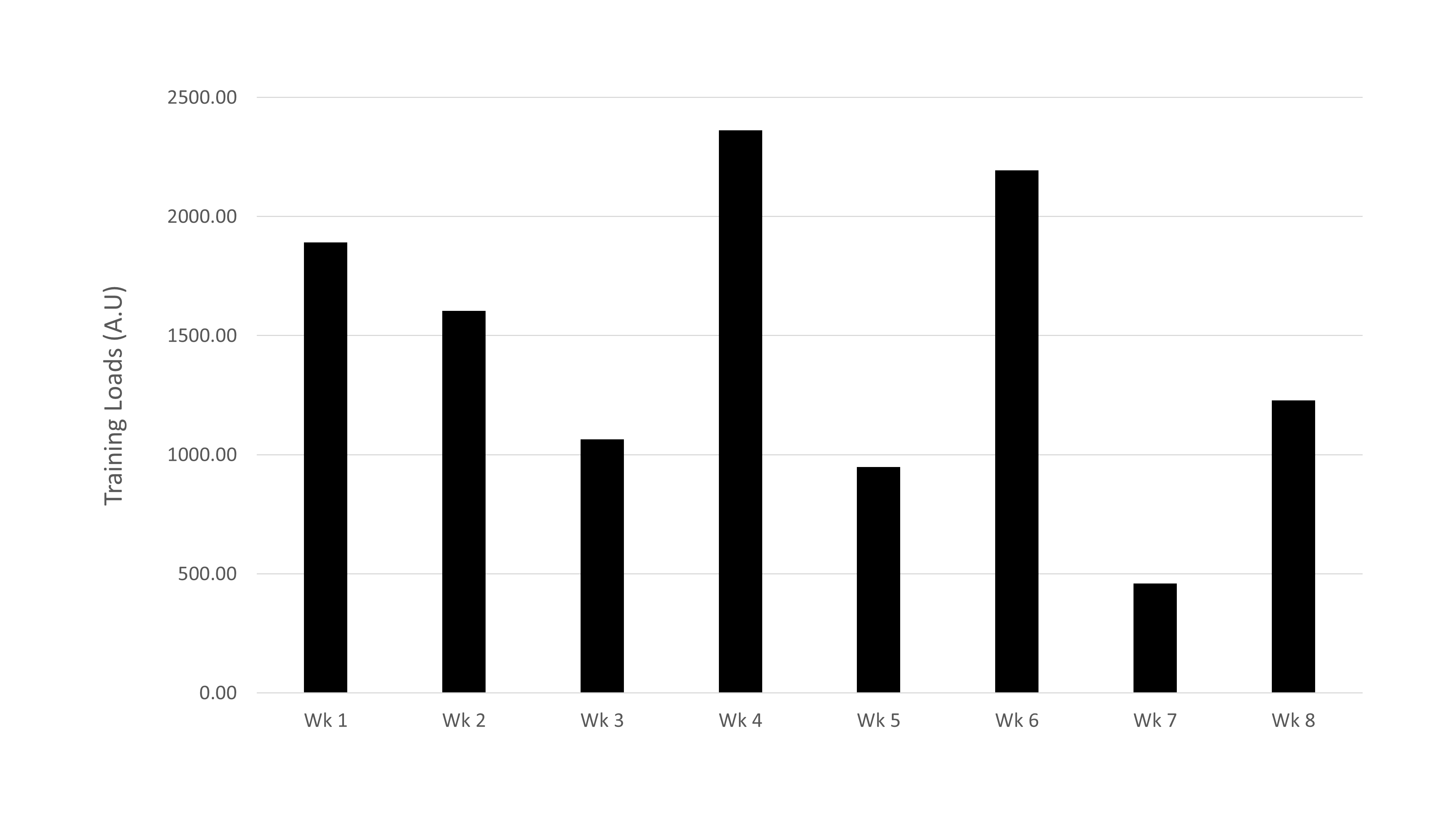

Supplement: Supplementary file 1 [file sports-09-00094-s001.zip › sports-1197701-SM/Figure S7 TL P7.tif]

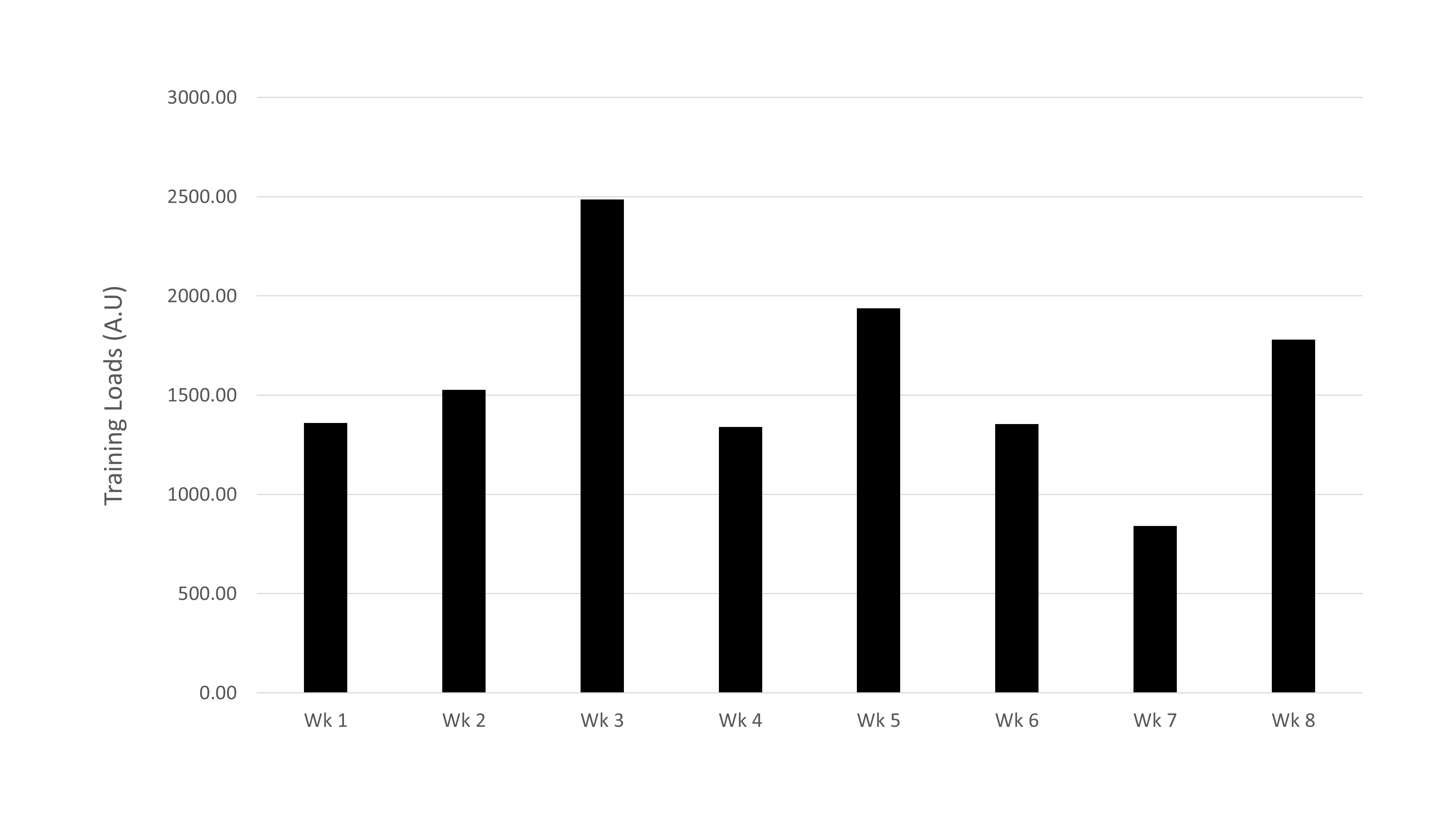

Supplement: Supplementary file 1 [file sports-09-00094-s001.zip › sports-1197701-SM/Figure S8 TL P8.tif]

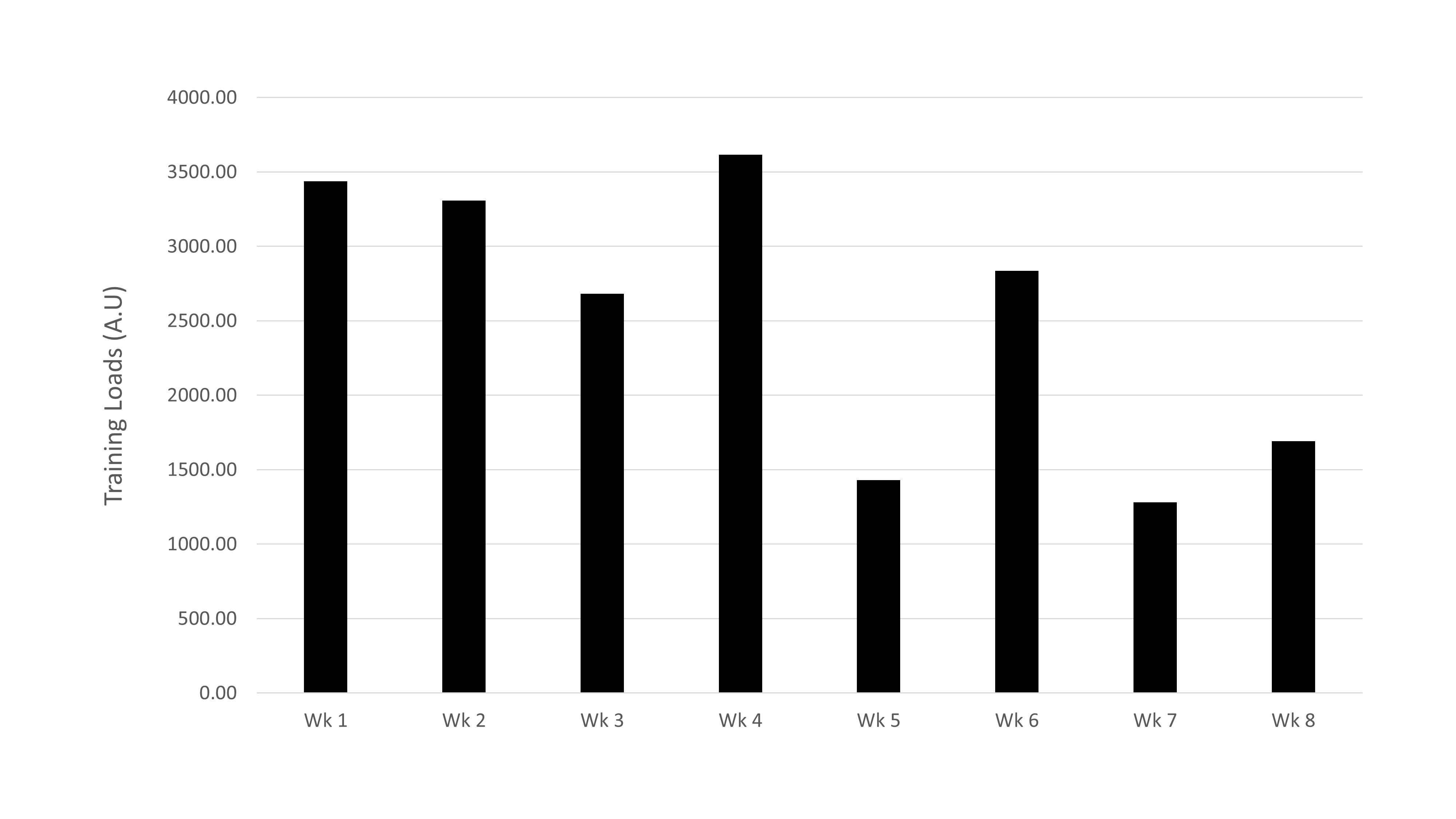

Supplement: Supplementary file 1 [file sports-09-00094-s001.zip › sports-1197701-SM/Figure S9 TL P9.tif]
